# Ageing and Obesity Shared Patterns: From Molecular Pathogenesis to Epigenetics

**DOI:** 10.3390/diseases9040087

**Published:** 2021-11-29

**Authors:** Abdelaziz Ghanemi, Mayumi Yoshioka, Jonny St-Amand

**Affiliations:** 1 Functional Genomics Laboratory, Endocrinology and Nephrology Axis, CHU de Québec-Université Laval Research Center, Québec City, QC G1V 4G2, Canada; abdelaziz.ghanemi@crchudequebec.ulaval.ca (A.G.); mayumi.yoshioka@crchudequebec.ulaval.ca (M.Y.); 2 Department of Molecular Medicine, Faculty of Medicine, Laval University, Québec City, QC G1V 0A6, Canada

**Keywords:** obesity, ageing, epigenetics, pathogenesis, exercise

## Abstract

In modern societies, ageing and obesity represent medical challenges for healthcare professionals and caregivers. Obesity and ageing share common features including the related cellular and molecular pathways as well as the impacts they have as risk factors for a variety of diseases and health problems. Both of these health problems also share exercise and a healthy lifestyle as the best therapeutic options. Importantly, ageing and obesity also have common epigenetic changes (histone modification, DNA methylation, noncoding RNAs, and chromatin remodeling) that are also impacted by exercise. This suggests that epigenetic pathways are among the mechanisms via which exercise induces its benefits, including ageing and obesity improvements. Exploring these interrelations and based on the fact that both ageing and obesity represent risk factors for each other, would lead to optimizing the available therapeutic approaches towards improved obesity management and healthy ageing.

## 1. Biological Similarities between Ageing and Obesity

Ageing and obesity are major topics in biomedical studies, mainly because both represent risk factors for numerous diseases and health conditions [1]. The modern lifestyle and industrial development have increased obesity rates as well as the aged population percentage worldwide. Obesity is specifically increasing among the elderly [2,3], which contributes to sarcopenic obesity, a chronic, age-related class of obesity [4,5]. These interactions between two important risk factors strengthen the need to further deepen our biological and clinical understanding of the interrelation and correlations between ageing and obesity. Such mechanistic elucidation would allow to develop the medical care including geriatrics and obesity management, among other chronic diseases. Within this piece of writing, we aim to elucidate selected links between ageing and obesity through different illustrations starting from pathogenesis and molecular pathways towards epigenetics, supported by evidences from exercise being a therapeutic tool for both.

Obesity is defined as an abnormal accumulation of adiposity resulting from a disturbed energy balance in which energy intake is higher than energy expenditure [6] with a modified metabolic phenotype [7], complex neuroendocrine changes [8], and pathogenic implications [9]. Obesity has even been classified as a disease [10] and associated with health problems including impaired fertility [11,12], neurodegenerative disease [13], cognitive decline (in mid-life) [14], coronavirus disease 2019 (COVID-19) severity and resulting health problems [15,16,17,18], type 2 diabetes [19], cancer [20], cardiovascular diseases [21], pulmonary diseases [22], insulin resistance [23], atherosclerosis [24], mitochondrial dysfunction [25], dyslipidemia [26], liver disease [27], impaired immunity [28,29], and impaired regeneration [30]. Ageing, on the other hand, represents the progressive decline of the biological functions with time [31]. It also represents a risk factor for numerous diseases and health conditions, many of which are similar to those associated with obesity. These include neurodegenerative disease [32], cognitive decline [33], COVID-19 severity [34], type 2 diabetes [35], skeletal muscle loss [36], cancer [37], cardiovascular disease [38], pulmonary disease [39], insulin resistance [40], atherosclerosis [41], mitochondrial dysfunction [42], dyslipidemia [43], liver disease [44], fertility alteration [45,46] immunity alteration [47], and declined regeneration [48].

Although the risks related to obesity are independent from ageing and those related to ageing are independent from obesity, such similarities between ageing and obesity as risk factors suggest common patterns and share underlying mechanisms of both ageing and obesity. Early epidemiologic data approved the prevalence of obesity increases by ageing, especially in women. Therefore, the ongoing step is to know more about how ageing and obesity could be related at the molecular level. Within this context, obesity and ageing have been described as sharing common pathways at the molecular and cellular levels. For instance, in both, we have increased inflammation [49,50], free radicals, and oxidative stress [51,52] as well as microbiota changes [53,54]. In addition, healthy diet and physical activity are prescribed to manage obesity [55,56] and also optimize healthy ageing [57,58]. While the main goal of prescribing the physical activity in obesity is to increase the energy expenditure and, thus, reduce the adiposity and lose weight [59,60], in ageing, the physical activity aims mostly to improve muscular and metabolic performance [57,61,62]. Importantly, physical activity as a common therapy for both ageing and obesity has significant impacts on reducing the risk factors mediated by ageing and obesity and also improves numerous biomolecular markers and pathological profiles. As illustrations, physical activity improves and optimizes treatment/prevention or reduces the risk of metabolic disorders [63], cancer [64], cardiovascular disease [65,66,67], immune functions [68], insulin resistance [69], oxidative stress [70], liver disease [71], regeneration [72,73], pulmonary disease [74,75], atherosclerosis [76], and mitochondrial remodeling [77]. These evidences add up on those of functional genomics [78,79,80,81] as illustrated by the secreted protein acidic and rich in cysteine (SPARC). Indeed, SPARC expression changes during obesity [82] and with ageing [83] and *Sparc/SPARC* represents an exercise-induced gene upon which exercise-induced muscle phenotype changes would depend [84,85]. In addition, SPARC is involved in diverse biological activities [86] related to those described above in the context of obesity, ageing, and exercise. These include metabolic and homeostatic properties [87], inflammation [88], cancer [89], regeneration [90], and metabolism [91]. This exercise-induced key myokine with obesity and age-related expression patterns further points to molecular links between obesity and ageing.

## 2. Epigenetics: An Additional Link between Ageing and Obesity

Furthermore, epigenetics studies provide additional evidences of similar patterns shared by obesity and ageing. Therefore, epigenetics represents a field worth exploring to reveal further links between obesity and ageing. This is reflected by the changes such as histone modification, DNA methylation, noncoding RNAs, and chromatin remodeling that have been associated with both ageing [31,92,93,94,95,96,97] and obesity [98,99,100,101,102,103]. These changes can follow diverse patterns. For instance, region-specific DNA hypermethylation [104] and proliferation-dependent alterations of the DNA methylation [105] have been reported in ageing during which we talk about epigenetic clocks [106]. The possible use of DNA-methylation-based measures as a tool to evaluate the accelerated biological ageing [107,108,109,110] represents a potential application of the DNA methylation age (DNAmAge), which would contribute to several diseases such as obesity. Similarly, obesity-related DNA methylation can be site-specific [111] and with specific methylation signatures [112]. Other related features such as telomere attrition are also shared between ageing [31] and obesity [113,114]. 

Importantly, exercise—prescribed for both elderly and obese patients—has impacts on the epigenetics patterns related to both ageing and obesity including DNA methylation [115], histone modification [116], chromatin modifications [117], and noncoding RNAs [118]. These exercise-related properties suggest that epigenetics pathways are among the mechanisms via which exercise induces its benefits—as it has been shown, for instance, for exercise-mediated heart protection [119]. They further support targeting epigenetic pathways as a therapy [120,121] as well; potentially, to treat obesity and improve ageing. These observations also suggest correlations between epigenetics changes and obesity/ageing-related pathologic phenotypes. In addition, these molecular and clinical evidences, from genetics to epigenetics and pathogenesis, further present obesity as a risk factor for ageing and, at the same time, highlight ageing as a risk factor for obesity [1]. This would explain why losing weight “rejuvenates”. Moreover, dietary restriction (that has both antiageing and antiobesity effects) also impacts epigenetics towards significant health benefits [122,123] bringing an additional correlation between ageing and obesity.

## 3. Perspectives

These introduced concepts would have important applications in the medical fields, especially that both ageing and obesity are among what medically characterize the epidemiological and pathological profiles of most modern societies. Although a healthy ageing is the optimum target of geriatrics, we have limited options to manage ageing (irreversible time effects). However, obesity, on the other hand, has realistically more management options since it remains relatively reversible. Therefore, managing obesity toward healthy ageing remains more practical than targeting healthy ageing to manage obesity. It is worth noting, however, that treating obesity would optimize ageing and healthy ageing would decrease obesity risk. Nevertheless, the key approach remains to target a healthy lifestyle including exercise, diet, sleeping, and psychological well-being to manage obesity, optimize healthy ageing, and control most diseases’ risk factors. 

**Figure 1:**
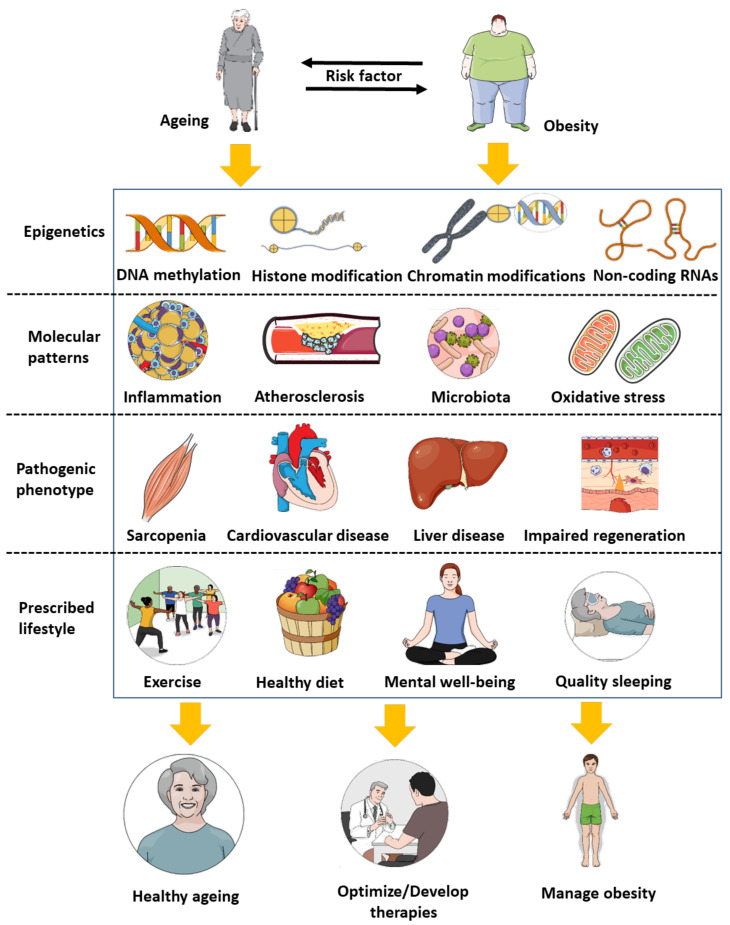
Examples of ageing and obesity shared patterns. Both ageing and obesity represent a risk factor foreach other. Elucidating the patterns shared between ageing and obesity, from epigenetics to molecular pathogenesis, would allow to both optimize healthy ageing and manage obesity.

We would like to introduce a new concept via which there is a potential to combine the age-related and the obesity-related epegentics measures to obtain a full evaluation of how deep both the age and obesity worsen the other as well as the various diseases and risk factos for which either ageing or obesity represent a risk factor. The need to actualize this idea nowadays comes from the urgent epedemiological situations related to ageing and obesity in the modern societies both in developed and in developing countries. To expand this vision, the advances and added value of this work is that it puts epigenetics along with pathological phenotype, molecular patterns, and lifestyle impacts as a set that regroups the elements shared between obesity and ageing (Figure 1). Such approches would allow for optimizing therapies and lifestyle management choices.

## Data Availability

Not applicable.
